# The long and the short of it: the MDM4 tail so far

**DOI:** 10.1093/jmcb/mjz007

**Published:** 2019-03-28

**Authors:** Sue Haupt, Javier Octavio Mejía-Hernández, Reshma Vijayakumaran, Simon P Keam, Ygal Haupt

**Affiliations:** 1 Tumor Suppression Laboratory, Peter MacCallum Cancer Centre, 305 Grattan Street, Melbourne, Victoria, Australia; 2 Department of Clinical Pathology, The University of Melbourne, Parkville, Victoria, Australia; 3 Department of Biochemistry and Molecular Biology, Monash University, Clayton Campus, Victoria, Australia; 4 Sir Peter MacCallum Department of Oncology, The University of Melbourne, Parkville, Victoria, Australia

**Keywords:** MDM4, MDMX, MDM4-FL, MDM4-S, p53, MDM2, cancer

## Abstract

The mouse double minute 4 (MDM4) is emerging from the shadow of its more famous relative MDM2 and is starting to steal the limelight, largely due to its therapeutic possibilities. MDM4 is a vital regulator of the tumor suppressor p53. It restricts p53 transcriptional activity and also, at least in development, facilitates MDM2’s E3 ligase activity toward p53. These functions of MDM4 are critical for normal cell function and a proper response to stress. Their importance for proper cell maintenance and proliferation identifies them as a risk for deregulation associated with the uncontrolled growth of cancer. MDM4 tails are vital for its function, where its N-terminus transactivation domain engages p53 and its C-terminus RING domain binds to MDM2. In this review, we highlight recently identified cellular functions of MDM4 and survey emerging therapies directed to correcting its dysregulation in disease.

## MDM4 regulation of p53

The pioneering discovery of MDM4 was made in mice by the Jochemsen group (Shvarts et al., 1996). Its human orthologue is referred to as HDM4, but for ease, in this review, we will use a generic ‘MDM4’ without distinction. MDM4 was identified by its engagement with the major tumor suppressor protein p53 and named through its structural similarity with MDM2; with the two forming a family (Shvarts et al., 1996). p53 is a transcription factor that acts as a fundamental determinant of cell fate in response to cellular stress (Kastenhuber and Lowe, 2017). p53 activities were originally shown to be strictly controlled by its major E3 ligase MDM2 (Haupt et al., 1997; Kubbutat et al., 1997; Honda and Yasuda, 2000). MDM4 was initially identified to influence p53 transcriptional activity (Shvarts et al., 1996). Evidence that MDM4 also critically influences p53 function during development was provided by the demonstration that its absence causes embryo lethality, which is rescued by elimination of p53. These studies elucidated that during development, proper regulation of p53 activity requires MDM4 (Parant et al., 2001; Finch et al., 2002; Migliorini et al., 2002), separate from its vital dependence on MDM2 E3-ligase activity (Jones et al., 1995).

In the human genome, *MDM4* is encoded at chromosome 1q32.1 and is comprised of 11 exons that are expressed as a number of isoforms (elaborated in Section ‘*MDM4 isoform splicing*’). Four key domains are conserved in the MDM proteins and their chronological order in human MDM4 is an N-terminus that binds to the major tumor suppressor p53, an acidic region, a zinc finger region, and a C-terminal RING (Tan et al., 2017). MDM4 shares high sequence homology with its family member (~55%) (Chen et al., 2012), but has the important distinction that its RING motif lacks appreciable E3-ligase activity toward p53, in contrast to the potent activity of MDM2 (Iyappan et al., 2010 and references within). How it intervenes to regulate p53 is examined in this review.

A single MDM protein has been traced back to ancient eukaryotes, with the appearance of the distinct lineages of closely related MDM2 and MDM4 arising around the time of vertebrate emergence, in a likely gene duplication event (Tan et al., 2017). The parallel ancient emergence of *TP53* and ancestral *MDM* in primitive DNA (Chakraborty et al., 2015) is predictive of elemental functions that benefit from methodical regulation, as is preserving essential DNA code. Dysregulation of MDM4 in cancer has drawn significant attention over the last two decades and now fascinating recent findings indicate that its influence extends well beyond this context. In this review, we will focus on new functions of MDM4, dictated by both the N- and C-tails respectively of this molecule.

### Wild-type p53

**Figure 1:**
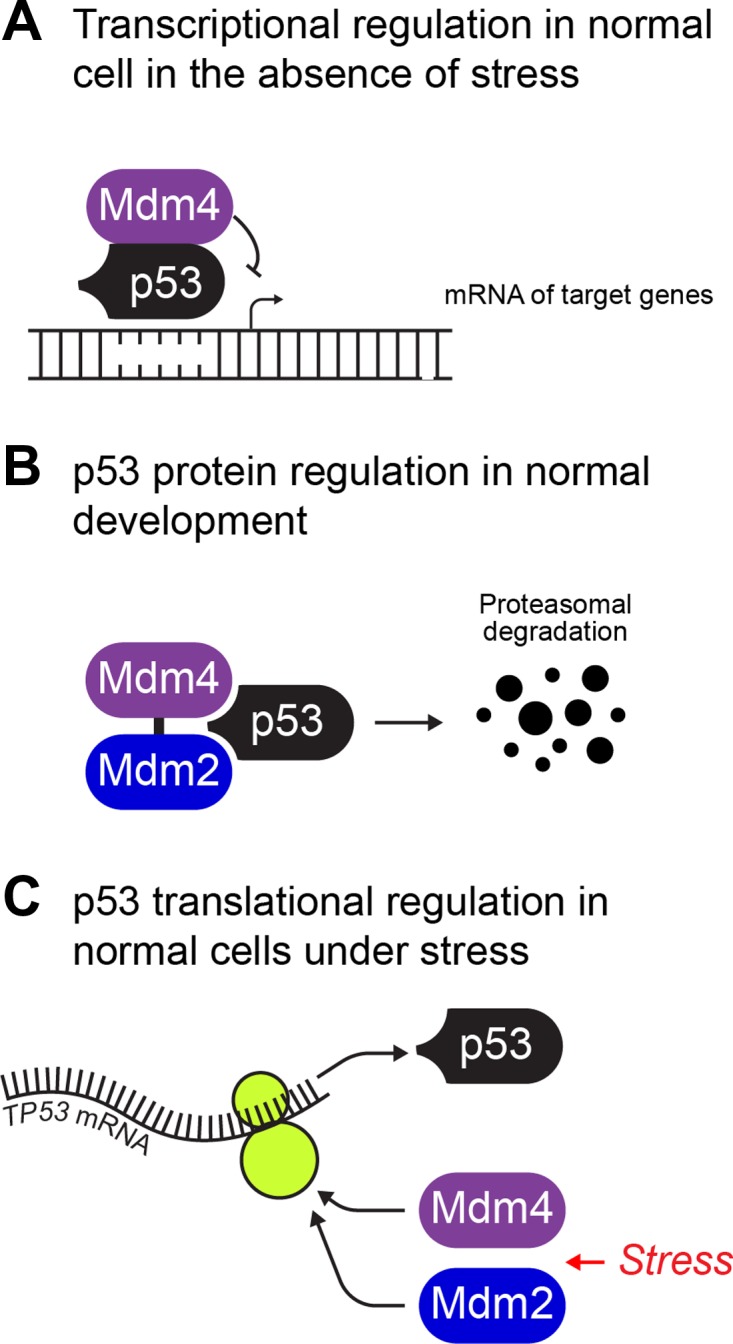
MDM4 regulates p53 at three key levels. (**A**) MDM4 binds to wt p53 and inhibits its transcriptional activity in normal cells and when MDM4 is elevated in cancers. (**B**) MDM4 promotes MDM2 E3 ligase activity towards p53 during development. (**C**) MDM4 and also MDM2 promote p53 translation from its IRES in response to stress.

MDM4 critically regulates p53 across three fundamental levels (Figure 1). The potency of unleashed p53 to impose growth restriction (Bieging et al., 2014) requires that it is kept incapacitated under normal conditions and MDM4 is instrumental in this restraint. Reciprocally, when p53 activation is required, MDM4 releases its control. For healthy cell viability, this regulation by MDM4 must be tightly and dynamically regulated. If these constraints over p53 transcriptional activity are not properly managed, MDM4 activity can become oncogenic as we will discuss in Sections ‘**Regulation of MDM4 in health and disease**’ and ‘**Oncogenic MDM4 functions**’.

Firstly, MDM4 inhibits p53 transactivation capacity through direct protein–protein engagement (Figure 1A). This occurs under physiological conditions, in an MDM2-independent manner (Francoz et al., 2006). Three domains of MDM4 engage p53 to achieve this inhibition. Respectively, MDM4 binds through its N-terminus (across residues 19–102) to the p53 N-terminal transcription activation domain, with its key contacts at hydrophobic residues PHE19, TRP23, and LEU26 (Popowicz et al., 2008).

In addition to this primary (canonical) MDM4 N-terminal engagement, a secondary interaction is established between the MDM4 acidic domain and the p53 DNA binding domain, which is inhibitory to p53 function. MDM4 Ser289 phosphorylation, catalyzed through CK1α engagement is critical for this secondary interaction. The proposed model predicts that MDM4 Ser289 phosphorylation frees the MDM4 acidic domain from its own C-terminal RING region, allowing this acidic domain to engage the internal p53 DNA binding domain. Under physiological conditions *in vivo*, this mechanism keeps p53 in check, curbing its transactivation function and its tumor suppressor capacity (Huang et al., 2018). A third interaction site is located at the MDM4 Zinc finger domain, where Ser314 phosphorylation stabilizes the MDM2–MDM4 complex and suppresses p53 transactivation activity. Tyrosine kinase signaling cascades involving AXL (de Polo et al., 2017) and ErbB4 (Gerarduzzi et al., 2016), dictate these particular post-translational phosphorylation events (as elaborated in Section ‘*Ubiquitination*.’). Vitally, in response to stress, p53 must be relieved from MDM4 engagement and this is also tightly regulated by post-translational modifications. Deregulation of these kinases with consequent compromised p53 activity pose risks in a number of cancer types (de Polo et al., 2017).

Secondly, MDM4 acts with MDM2 to promote p53 degradation (Figure 1B). MDM4 and MDM2 engage through their C-terminal ring domains (Tanimura et al., 1999) and through this heterodimerization MDM4 facilitates MDM2 to degrade p53 (Uldrijan et al., 2007; Wang and Jiang, 2012). Specifically, their engagement stabilizes MDM2 by inhibiting its auto-ubiquitination and in turn potentiating its E3 ligase activity toward p53 (Jackson and Berberich, 2000; Gu et al., 2002; Okamoto et al., 2009). This is fine-tuned by their relative stoichiometry (Poyurovsky et al., 2007). The absolute requirement for concerted MDM2–MDM4 regulation of p53 was most clearly demonstrated during early mouse development; although, at least in mice, MDM4 did not prove essential for controlling MDM2-regulation of p53 during normal, later development (Pant et al., 2011).

Thirdly, MDM4 promotes p53 protein synthesis in response to genotoxic stress activation (Figure 1C), arising from double-strand DNA breaks. Stress-induced p53 synthesis involves translation from its mRNA internal ribosome entry site (IRES) and the process is activated by IRES trans-acting factors (ITAFs). MDM4 and MDM2 act as non-redundant ITAFs that synergize to promote p53 expression. Specifically, the sequential process induced by DNA-damage (Doxorubicin) initiates MDM4 phosphorylation at Ser403, catalyzed by ataxia telangiectasia mutated (ATM), a serine/threonine kinase and a master cell cycle checkpoint regulator, which also phosphorylates MDM2 at Ser395 in parallel (but distinctly responds to ionizing radiation, as described in Section ‘*Phosphorylation/dephosphorylation*.’). The MDM4 C-terminal Ring in turn engages nascent *p53* mRNA, altering its structure to allow access for *p53* mRNA–MDM2 interaction, which in turn drives p53 synthesis. MDM4 was demonstrated to engage *p53* mRNA at its nucleotides encoding codons 10, 21, and 22. The Fahraeus group who undertook this elegant study, made the fascinating prediction that the unexpectedly high frequency of ‘silent p53 mutations’ at these sites are selected in disease due to their ability to hinder the MDM4–*p53* mRNA interaction and prevent proper stress-activation (Malbert-Colas et al., 2014).

This work also exposes essential levels of dependency between MDM4 and MDM2 for coordinated regulation of p53 synthesis, to establish an appropriate response to stress. This pioneering work provides clarity to the dogma that p53 mRNA levels do not dramatically alter in response to stress (Ponnuswamy and Fahraeus, 2012). These studies identify p53 translation as a chronologically early response to increase levels of newly synthesized p53 protein, in a process critically regulated by MDM4, in conjunction with MDM2 (Malbert-Colas et al., 2014). This does not contradict the existing understanding that p53 post-translational modifications promote its stress-induced stability (Kastenhuber and Lowe, 2017), or that overall *p53* mRNA levels are relatively unaltered by stress; but rather, these new findings suggest an additional vital level of control (Malbert-Colas et al., 2014). This work predicts that under stress, there is a relocation of the site of protein manufacturing (without noticeable increase in productivity) and in turn, the newly synthesized protein product becomes stabilized by the addition of post-translational modifications (as outlined in Section ‘*MDM4 protein structural modifications and implications for stability and function*’).

These complex levels of MDM4 involvement in p53 regulation raise fascinating questions regarding the ancestral genetic divergence that divided the E3 ligase competent MDM2, from the E3 ligase inactive MDM4 and in the process added to the finer regulation of p53 at multiple levels. Primarily, it raises questions regarding whether the emergence of more complex biological systems, requiring more sophisticated genomic protection, as offered by p53, require a more tightly controlled regulation process as offered by the MDM protein family. Also, it will be fascinating to understand how MDM4 and MDM2 interplay in elephants, which have ~20 copies of *TP53* and extended longevity in the absence of cancer (Sulak et al., 2016).

### Mutant p53

MDM4 was demonstrated to interact through its N-terminus (independent of its C-terminal RING), not only with wild-type (wt) p53, but also with mutant p53, as decisively identified in tissues from a knock-in mutant p53 mouse model (tp53^R172H^, the equivalent of human hotspot p53^R175H^; Pant et al., 2011). Whether mutant p53 levels are subject to regulation by MDM4 in conjunction with MDM2 at an early stage of cancer onset, is yet to be demonstrated. Notably, p53 mutation and elevated MDM2 levels are rarely coincident in tumors (at least in sarcomas) (Oliner et al., 1992). This predicts that MDM4 is unlikely to impact on mutant p53 levels, in the absence of MDM2 E3 ligase activity in a proteasome-mediated response. However, our findings demonstrate that oncogenic MDM4 promotes cancers driven by mutant p53 (Miranda et al., 2017) (as we discuss in Section ‘**Oncogenic MDM4 functions**’).

### p53 family members

MDM4 not only engages p53 but it also binds to the p53 family members p73 (Ongkeko et al., 1999) and p63. Intriguingly, MDM4 binding affinity with p63 and p73 is higher than the affinity of the family members respectively with MDM2 (Zdzalik et al., 2010). In contrast to the developmental capacity of p53 to rescue MDM4 null embryonic lethality however, p73 loss is incapable of a comparable rescue. Further, p53 appears to dominate biological outcomes associated with altering the MDM4–p73 axis, as demonstrated in a mouse tumor model (Tashakori et al., 2016). Whether the MDM4–p73 axis becomes significant in a cancer context, where normal p53 function is compromised, will be interesting to address in future studies.

## Regulation of MDM4 in health and disease

MDM4 gained its reputation as a negative regulator of wt p53, with oncogenic potential in the instance of its dysregulation (Section ‘**Oncogenic MDM4 functions**’). In more recent studies, however, evidence has emerged that it can be oncogenic independent of wt p53: in the absence of p53 and also in the context of mutant p53. Importantly, *MDM4* mRNA levels are elevated in numerous cancers, and while altered copy number is responsible for this in some cancers (Marine and Jochemsen, 2016), altered post-transcriptional events have emerged in others. In this section, we inspect determinants of MDM4 levels, other than copy number, and the disease risks associated with this dysregulation.

### MDM4 and single nucleotide polymorphisms (SNPs)

The relevance of MDM4 SNPs to disease is beginning to emerge as we discuss in this section in the context of cancer and viral susceptibility.

#### MDM4 SNPs and cancer risk

A number of SNPs in MDM4 have been linked with altered cancer susceptibility. Of particular interest is the rs4245739 A > C polymorphism, which is located at 1q32, in the 3′-untranslated region (UTR) of *MDM4*, 32 bp downstream of the gene. Notably, the C-allele is linked to cancer resistance, while the A-allele is defined as the risk allele for a number of cancers: ovarian (Wynendaele et al., 2010), prostate (Eeles et al., 2013), and breast (Garcia-Closas et al., 2013). A fascinating mechanism involving microRNA (miR) targeting has emerged to dictate these alternative fates (miRs are discussed in greater detail in Section ‘*miRs that regulate MDM4*’). The C-allele creates a putative novel binding site for three miRs, miR-191 (Wynendaele et al., 2010; McEvoy et al., 2012), miR-887, and miR-3669 (Stegeman et al., 2015), and correlates with low levels of MDM4. Unexpectedly, these miRs do not alter *MDM4* mRNA levels but instead, dictate the efficiency of its translation, with the A-allele being permissive and the C-allele being inhibitory (Stegeman et al., 2015). These findings are consistent with high MDM4 levels having oncogenic function in ovarian (Wynendaele et al., 2010) and prostate (Stegeman et al., 2015) cancers, and expand the studies of the first MDM4 SNPs identified in breast and ovarian cancers (Atwal et al., 2009).

#### MDM4 SNPs and viral susceptibility

Intriguingly, MDM4 has been demonstrated to have potent anti-viral properties, which have been linked to its SNPs. In the context of Human Papilloma Virus 16 (HPV16), MDM4 SNP variants SNPs (rs11801299 G > A, rs10900598 G > T: both located in the 3′-UTR region; and rs1380576 C > G: in intron 1) have been linked to increased risk of squamous cell cancer of the oro-pharynx (SCCOP) (Yu et al., 2012). This is particularly evident in non-Hispanic, non-smokers and non-drinkers. In this disease context, the negative impact of MDM4 on p53 transcriptional activity is speculated to be further compounded by HPV E6-mediated promotion of p53 proteasomal degradation (Lu et al., 2017). Pertinently, the SCCOP subset of SCC of head and neck (SCCHN) is growing in incidence, despite reduced smoking frequency and HPV infection is now recognized as a principle cause of this increase. However, only a fraction of HPV-infected people develop SCCOP, which led to a search for additional contributing elements (Lu et al., 2017), which uncovered this MDM4 link (Yu et al., 2012).

It will be fascinating also to determine whether the capacity to resist adenoviral replication that has been associated with MDM4 is also SNP-dependent. Where in the instance studied, reduction of MDM4 levels by the virus appeared to be at least partially independent on the proteasome (Yang et al., 2012). Equally intriguing would be the identification of whether MDM4 SNPs affect neuronal damage in HIV-associated neurocognitive disorders (HAND). Notably, while MDM4 has been identified to perform a pro-survival function in neurons, its levels are reduced in the mid-frontal cortex of patients with HAND. MDM4 is defined as a direct calpain substrate (Colacurcio et al., 2013) and intriguingly, HIV Tat activates calpain proteases (Perry et al., 2010).

### Regulation of MDM4 transcription

In humans, MDM4 has two promoter regions, referred to as P1 and P2. Transcription initiation from P1 is constitutive and results in a 75-kDa MDM4 isoform, this appears to be involved in maintaining low levels of p53 activity in healthy proliferating cells. Transcription initiation shifts to the second promoter P2, to preferentially drive the expression of a 76-kDa isoform of MDM4, in a cell-type and stress specific manner. Pertinently, a functional p53 responsive element was identified in the P2 promoter and chromatin-immunoprecipitation confirmed direct p53 engagement at this p53 response element. The distinct molecular weight of these isoforms reflects inclusion of an additional 18 amino acids in the N-terminus of the 76-kDa isoform. Specifically, P1 and P2 mRNA products differ in the inclusion of distinct 5′-ends of exon 1 or exon 1β in the first intron, respectively. Relevantly, the *MDM4* mRNA expressed from the P2 promoter was more efficiently translated than its mRNA counterpart generated from the constitutive P1 promoter (Phillips et al., 2010). This has functional implications for p53 activity as discussed in Section ‘*MDM4 isoform splicing*’.

*MDM4* expression is also directly induced by expression of the estrogen receptor α (ERα) (Swetzig et al., 2016). The scale of this risk is evident in breast cancer where ~65% of all cases overexpress ERα (Jiang et al., 2012). Notably, although this regulation is p53-independent, the outcome of overexpression of oncogenic MDM4 poses both p53-independent and wt p53-dependent risks (Swetzig et al., 2016) (as reviewed in Section ‘**Oncogenic MDM4 functions**’).

Mitogenic signaling, through the Ras-MAPK pathway is linked to elevated *MDM4* expression levels, at least in some cancers e.g. breast cancer line MCF-7. The MDM4 promoter was identified to contain binding sites for Ras downstream targets, the Ets-family of transcription factors (c-Ets-1 and Elk-1), and their engagement corresponds with increased *MDM4* expression. Consistently, MDM4 levels were elevated in response to activated K-Ras and insulin-like growth factor 1 (Gilkes et al., 2008). This activation pathway appears specific to certain cancers, for example BRAF and NRAS status was not found to correlate with MDM4 levels in melanoma (Marine and Jochemsen, 2016).

### MDM4 isoform splicing

MDM4 expression is undetectable in most normal, unstressed, adult tissues (Dewaele et al., 2016), but not all, as we showed in normal adult, female breast ducts (Haupt et al., 2015) and as was also shown in brain, colon and thymus (De Clercq et al., 2010). In contrast, its RNA transcripts appear to be ubiquitously expressed (Parant et al., 2001). MDM4 abundance has been attributed to the stability of its RNA isoforms (Bezzi et al., 2013; Bardot et al., 2015; Dewaele et al., 2016). MDM4 isoforms are generated by alternative pre-RNA splicing and also by post-translational modifications, including phosphorylation and ubiquitination (Colacurcio et al., 2013). Dysregulated MDM4 splicing has been linked to cancer, but not without controversy.

Two major *MDM4* mRNA isoforms have been reported. Stable *MDM4-full length* (*MDM4-FL*) results from the inclusion of exon 6. Unstable *MDM4-short* (*MDM4-S*) excludes exon 6. The generation of these distinct isoforms involves a number of splicing factors (Bezzi et al., 2013; Dewaele et al., 2016). A third form MDM4^p60^ has also been reported (Tournillon et al., 2017). At least four additional RNA isoforms have been identified, but it is unclear whether these are translated to protein. We refer to the extensive analysis of these isoforms as previously reviewed (Mancini et al., 2009a) and focus here on recent findings.

#### MDM4-S

The absence of detectable MDM4 from most adult tissues has been attributed to the preferred production of the unstable isoform MDM4-S (Dewaele et al., 2016). However, the roles of MDM4-S have been shrouded in ambiguity. Mechanistically, *MDM4-S* is generated by an exon-skipping event, which results in the introduction of a stop codon at residue 127. A 17 kDa protein is predicted from *MDM4-S* (Rallapalli et al., 1999). Vitally though, the *MDM4-S* transcript is unstable, due to its predisposition to nonsense mediated RNA decay (Bezzi et al., 2013; Bardot et al., 2015; Dewaele et al., 2016).

MDM4-S is comprised of the p53-binding domain, but lacks the central domains and the C-terminus. Over-expressed *MDM4-S* showed particularly strong p53 binding avidity (Rallapalli et al., 2003) and proved to be a more potent inhibitor of p53 function than *MDM4-FL*. Early reports identified that *MDM4-S* mRNA was only readily detected in rapidly dividing cells and transformed cells, where p53 growth inhibitory activities were efficiently suppressed (Rallapalli et al., 1999). Importantly though, MDM4 protein levels do not correspond with its RNA levels (Bardot et al., 2015).

#### MDM4-FL

Larger, stable isoforms of *MDM4* are generated by exon 6 inclusion in a process facilitated by the spliceosome component serine and arginine rich splicing factor 3 (SRSF3; a recognized oncogene). Notably though, SRSF3 is unable to mediate exon 6 inclusion unaided, possibly requiring additional splice factors (Dewaele et al., 2016). It is interesting then, that *MDM4* splicing is also impacted by the protein arginine methyltransferase 5 (PRMT5; Bezzi et al., 2013), which includes the spliceosome in its spectrum of protein targets (and notably also includes p53). PRMT5 methylation of the spliceosome is central in its assembly process and essential for splicing (Gerhart et al., 2018). Whether these processes are linked will be interesting to establish.

Predominantly, *MDM4-FL* mRNA is distinguished from *MDM4-S* mRNA, using PCR primers that straddle exon 6 (Dewaele et al., 2016). It is valuable to note that while this approach gives the impression that only two major MDM4 isoforms exist, additional isoforms not picked up by these primers have been identified to have biological significance. For example, two high MW MDM4 protein isoforms of 75 kDa and 76 kDa have been reported, but their extremely similar molecular weights do not allow for simple discrimination using the standard protein electrophoresis separation methods, and anti-MDM4 antibodies available. The size difference between these isoforms is attributed to initiation from distinct promoters referred to as P1 and P2 (Phillips et al., 2010), as described in Section ‘*Regulation of MDM4 transcription*’.

Of biological importance, the constitutive 75-kDa MDM4 isoform inhibits p53-dependent transcription (as demonstrated for *CDKN1A* [*p21*^*WAF1*^]), consistent with the requirement to keep p53 restrained in unstressed normal cells. On the other hand, in response to stress, the MDM4 76-kDa isoform (termed MDMX-L in the original reference) is preferentially transcribed and this isoform interferes with p53 engagement of the 75-kDa MDM4 isoform, in turn relieving its inhibitory effect on p53 transcriptional activity. Notably, in contrast to the 75-kDa isoform, the MDM4 76-kDa isoform does not inhibit p53 transcriptional activity efficiently. This 76-kDa isoform does, however, retain the capacity to bind to MDM2 and promote p53 ubiquitination, which in a timely manner promotes post-stress recovery, by returning p53 to its low basal levels, thereby entailing the MDM2–p53 negative feedback loop.

This potent capacity of particular MDM4 isoforms (i.e. the 75-kDa isoform) to attenuate p53 transcriptional activity (Phillips et al., 2010) also predicts a peculiar risk for cancer. It is worth noting that while particular interest has focused on distinguishing MDM4-FL from MDM4-S in cancers, such as those of the breast (Lenos et al., 2012), this work from the Jochemsen group (Phillips et al., 2010) would predict that more precise discrimination between the 75-kDa and 76-kDa forms in cancer would be extremely pertinent. Specifically, the 75-kDa isoform, which inhibits p53 transcriptional activity, would be predicted to pose a particular cancer risk in a wt p53 context. It will be interesting to identify whether the findings of the Jone’s lab that in some models MDM4 can actually promote genomic stability and suppress oncogenic cellular transformation (Matijasevic et al., 2016) are dictated by the promoter from which they arise. Whether the 76-kDa isoform can work in conjunction with MDM2 to ubiquitinate both wt and mutant p53 is a relevant open question. It raises questions of whether specific MDM4 promoters dominate in cancer and the ramifications for therapy.

#### Resolving the MDM4-S and MDM4-FL paradox

Ambiguity regarding the roles of MDM4 isoforms have arisen from a number of observations. First, an enhanced potency of MDM4-S to inhibit wt p53 activity (Rallapalli et al., 2003) was claimed to be a significant cancer risk (Rallapalli et al., 1999), which accounted for its high levels in numerous cancers (Bartel et al., 2005; Lenos et al., 2012; Liu et al., 2012). Second, it is unclear how MDM4-S could pose a significant cancer risk, as the *MDM4-S* transcript is unstable, due to its predisposition to nonsense mediated decay (Bezzi et al., 2013; Bardot et al., 2015; Dewaele et al., 2016). Third, *MDM4-FL* was identified as the major isoform form that arises from oncogene-driven mis-splicing (Dewaele et al., 2016).

To address these apparent anomalies, a number of mice models were generated with controlled *MDM4-S* expression. The Toledo group drew the conclusion that in response to stress, p53 becomes unfettered from MDM4-FL inhibition, through prioritized production of the unstable *MDM4-S*. To reiterate, *MDM4-S* appears to be generated as a regulatory byproduct, rather than representing an overt oncogenic risk (Bardot and Toledo, 2017). Independently, the Lozano lab demonstrated that elevated *MDM4-S* transcript levels in tumors arise from splicing defects, rather than being selected for inherent oncogenic capacity. Consistently, MDM4-S protein levels did not accumulate in their transgenic *MDM4-S* B-cell lymphoma mouse model (Pant et al., 2017). It must be noted that these findings have been generated in mice, and variation between the murine and human MDM4 homologes exists (Shvarts et al., 1996). Importantly though, these findings are in keeping with skin melanoma, where p53 is generally wild-type and *MDM4-FL* is generated at the expense of *MDM4-S* (Dewaele et al., 2016). They also are in agreement with the link of higher levels of the more stable *MDM4-FL* relative to *MDM4-S*, together with *TP53* exon mutation in metastatic breast cancer (Grawenda et al., 2015).

#### MDM4^p60^

A less studied MDM4 isoform of 60 kDa has also emerged that is N-terminal truncated and lacks the p53 binding domain, but includes the RING domain. MDM4^p60^ levels were noted for their peculiar capacity to dictate function in cells: where low levels drive MDM2 degradation; in contrast to higher levels that lead to MDM2 stabilization, while preventing MDM2 from degrading MDM4-FL protein (Tournillon et al., 2015).

An intriguing finding also from the group of Fahraeus is that generation of this isoform is favored in the context of hot spot p53 R273H DNA contact mutant, that lacks direct transactivation capacity as it is incapable of engaging p53 promoter responsive elements (Tournillon et al., 2017) (discussed further in Section ‘*Regulation of MDM4 translation*’).

### miRs that regulate MDM4

Post-transcriptional regulation of MDM4 RNA expression through complementary engagement with short non-coding RNA microRNAs (miRNAs, ~18–25 nucleotides in length of single-stranded RNA) is gaining recognition, with at least 7 targeting species identified since ~2010. miRs that target *MDM4* RNA can suppress cancer development through activation of wt p53 tumor suppressive function. In contrast, miR misregulation can have diabolical consequences for diseases such as cancer (Shen et al., 2018).

Notably, in normal human fetal retinae, miR-191 targeting of the *MDM4* 3′-UTR keeps MDM4 levels low. In contrast, retinoblastoma is linked to increased MDM4 levels that arise through protection from miR-191 targeting. In some retinoblastoma patient samples, the capacity of miR-191 to engage *MDM4* mRNA is eliminated by somatic mutations (SNPs) that eliminate this miR binding site (as discussed in Section ‘*MDM4 SNPs and cancer risk*.’); while in others miR-191 levels are lower than in the normal fetal retinae (McEvoy et al., 2012). A second miR to target the classic MDM4 3′-UTR region is miR-10a and its high expression levels are linked to acute myeloid leukemia (Ovcharenko et al., 2011). A third miR to target *MDM4* 3′-UTR is miR-370. Consistent with the theme of the other miR’s that target *MDM4*, colon cancer risk increases in an inverse correlation with miR-370 levels, and the converse is also true. Interestingly, this was evident in both wt and mutant p53 context, consistent with p53-dependent and independent oncogenic functions in this cancer-type (Shen et al., 2018). A fourth miR to target *MDM4* 3′-UTR is miR-766. Similarly, in cancer cells with wt p53, elevating miR-766 directly targets MDM4 and this in turn activates p53 (Wang et al., 2017).

miR-661 appears to be a primate-exclusive means of regulating both MDM2 and MDM4 expression, due to its homing to Alu elements (restricted to primates) in the 3′-UTRs of these genes. High miR-661 expression in wt p53 breast cancers consistently corresponds with better prognosis (Hoffman et al., 2014).

*MDM4* RNA is also targeted by miR-34a, where notably, its engagement occurs within the open reading frame of MDM4 exon 11, just up-stream of the classic 3′-UTRs miR targeting region. This site is identified to be common to all *MDM4* isoforms. In response to DNA damage (as induced by Doxorubicin treatment), *MDM4* RNA levels are lowered through miR-34a engagement. This effect is compounded by p53 inducing miR-34a expression. Abnormally low miR-34a expression (e.g. from a SNP in the miR binding site) and consequent MDM4 elevation, is a predicted cancer risk in a wt p53 context (Mandke et al., 2012 and references within).

In a cellular response to cisplatin, the involvement of MDM4 and miR-885-3p (Huang et al., 2011) and miR-1307 (Wang and Zhu, 2018) appears to be more complicated. In a positive response to cisplatin, miR-885-3p and MDM4 promote apoptosis. Specifically, cisplatin provokes miR-885-3p to bind to MDM4 5′-UTR mRNA and promotes MDM4 protein export from the cytoplasm into the mitochondria. In this context, MDM4 binds to p53 Ser46 and BCL2 (Huang et al., 2011). This parallels the original model the Moretti group proposed for a MDM4–p53 Ser46–BCL2 axis mediating apoptosis through the mitochondria, in response to the stress of cisplatin exposure. Specifically, they reported that MDM4 relocates to the mitochondria in response to stress, where it binds to BCL2 and in turn facilitates the engagement of BCL2 and promotes p53 Ser46 phosphorylation, with ensuing release of cytochrome C and apoptosis onset (Mancini et al., 2009b).

Alternatively, in a cisplatin-resistance breast cancer context, the levels of miR-1307 and its target MDM4 were identified to be decisive. Specifically, miR-1307 levels are low in breast cancer cells, while elevated levels of MDM4 are associated with cisplatin resistance (where both p53-dependent and independent activity is suggested). Increasing miR-1307 levels or reducing MDM4 levels each sensitize these resistant cancer cells to cisplatin (Wang and Zhu, 2018). These studies overall expose a critical role for miR regulation of MDM4 in dictating controlled cell growth and response to a standard of care cancer therapy with cisplatin. It will be fascinating to determine whether this extends to a full range of genotoxic drugs.

### Regulation of MDM4 translation

MDM4 translation can be regulated by both wt and mutant p53, but by different mechanisms. Wt p53 is described as a trans-repressor of MDM4. Specifically, wt p53 engages the 5′-UTR of *MDM4* mRNA and regulates its translation in a zinc-dependent manner. This synthesis process is controlled both through engagement of the p53 core and through additional involvement of the p53 N-terminal trans-suppression domain. This is reminiscent of a reciprocal mechanism identified for MDM4 in response to stress, however in that instance MDM4 promotes p53 translation from its IRES (Malbert-Colas et al., 2014) (as described by the same research team in Section ‘*Wild-type p53*’). These studies elaborate some of the intricacies of the dynamic loops involved in the p53–MDM4 regulation.

In contrast, mutant p53 has a distinct profile of *MDM4* mRNA engagement corresponding with an altered synthesis of MDM4 isoforms. Notably, in the context of p53 R273H mutant, MDM4-FL levels decreased, while MDM4^p60^ isoform levels increased. In the context of a p53 mutant that lacks specific DNA binding capacity, regulation at the translation level was deduced (Tournillon et al., 2017) (see also Section ‘*MDM4*^*p60*^.’).

### MDM4 protein structural modifications and implications for stability and function

**Figure 2:**
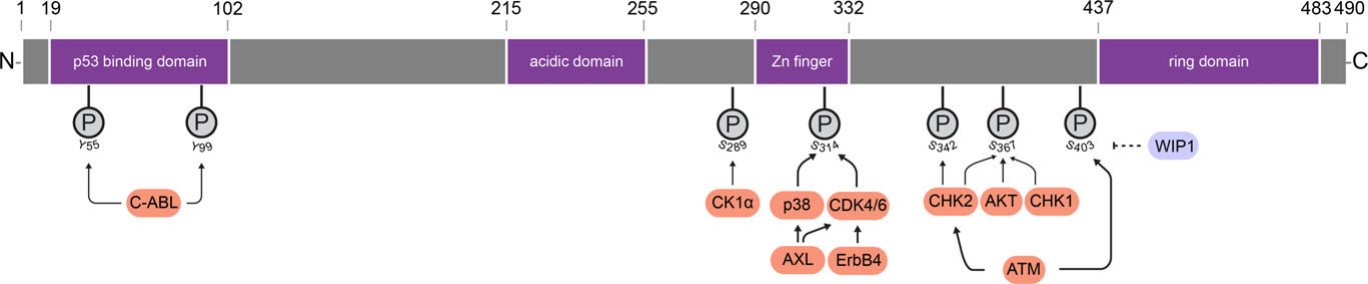
MDM4 structure is subject to extensive post-transcriptionally modification. Human MDM4 is comprised of an N-terminal p53-binding domain, a central domain with an acidic region and a Zn finger, and a C-terminal RING domain. MDM4 undergoes post-transcriptional phosphorylations, which are designated by ‘P’, at either tyrosine ‘Y’ or serine ‘S’. The specific kinases (orange color) and phosphatase (mauve color) that modify MDM4 dictate its activity temporarily and spatially.

MDM4 has strong structural similarities to its family member MDM2 (Shvarts et al., 1996), but it also has individual features that are not shared and these dictate its separate, unique, functional capacities. The post-translational modifications associated with MDM4 are vital for these functions (Figure 2), as we discuss in this section.

#### Phosphorylation/dephosphorylation

The tails of the MDM4 and MDM2 proteins (at least in humans and mice) are highly conserved: where their N-termini engage the tumor suppressor p53; and their C-termini contain the RING domains (Tan et al., 2017). Vital post-translational modifications imposed on the terminal regions in response to specific stimuli dictate MDM4 function. Identifying which MDM4 structural changes cause p53 release to consequently prime its tumor suppressor functions is highly important, not only for understanding the stress-activation process but also the subsequent steps of relief. These processes are also of potential relevance to revealing the mechanism of oncogenic MDM4 and MDM2 suppression of p53, and have obvious translational implications in targeted therapeutics. Kinases that modify MDM4 and consequently regulate p53 activity are visually summarized in Figure 2.

Our group studied the N-terminal phosphorylation of MDM4 by the tyrosine kinase C-ABL (or ABL1) and found that phosphorylation at Tyr99 interrupted its engagement of p53 (through steric clash with p53 Pro27), as required for p53 activation. A subsequent second phosphorylation at Tyr55, was then identified to restore MDM4–p53 binding, which facilitates recovery post-stress (Zuckerman et al., 2009). Molecular dynamic simulations elegantly elaborated these interactions further, predicting that the MDM4 N-terminus acts as a ‘lid’ whose phosphorylation status at these sites dictates its function: where phosphorylation of MDM4 Tyr99 ‘closes the lid on the p53-binding pocket’, and the following phosphorylation of MDM4 Tyr55 ‘opens the lid’ to expose the site for p53 interaction (Chan et al., 2017).

Additional kinases that phosphorylate MDM4 under defined conditions are elaborated in this section in the context of health and cancer risk. Importantly, different triggers provoke particular kinases, which dictate distinct outcomes: either p53 inactivation or activation. It appears likely that compounding modifications of MDM4 direct the specific outcomes (Lopez-Pajares et al., 2008).

In response to stresses, p53 becomes active when MDM4 is phosphorylated in its C-terminal region at residue Ser367 (LeBron et al., 2006). This in turn creates a binding site for adapter protein 14-3-3. The particular nature of the stress dictates the priming by distinct kinases. In response to UV damage (single DNA strand breaks), MDM4 is phosphorylated by CHK1 at Ser367, leading to MDM4 translocation to the cytoplasm, consistent with the activation of p53 (Jin et al., 2006). In contrast, in response to ionizing radiation, this process is driven through ATM (Pereg et al., 2005), which activates CHK2 to phosphorylate MDM4 at Ser367 and also Ser342 (Chen et al., 2005). In this instance, 14-3-3 binding induces MDM4 conformational change to expose a hidden nuclear location sequence. CHK2 and 14-3-3 co-ordinate to stimulate MDM2-mediated ubiquitination and degradation of MDM4, resulting in p53 activation (Okamoto et al., 2005; LeBron et al., 2006 and references within). Additionally, in response to severe DNA stress, in untransformed cells, cytoplasmic MDM4 dissociates from MDM2 and engages and stabilizes serine-threonine kinase HIPK2 to promote p53 Ser46 phosphorylation. This p53 phosphorylation event is associated with its nuclear translocation and transactivation activity. Notably this capacity was lost during cell transformation of breast cells, i.e. breast MCF10a vs. MCF7, respectively (Mancini et al., 2016).

Pertinently, p53 activation is averted by the activity of the phosphatase WIP1 (PPM1D). WIP1 suppresses phosphorylation at Ser367 indirectly. WIP1 dephosphorylates MDM4 Ser403 (Figure 2), which is the site of ATM phosphorylation and this in turn suppresses the phosphorylation of MDM4 Ser342 and Ser367. While this latter process is pertinent to resolving post-stress p53 levels, dysregulation of WIP1 is oncogenic, with clear risks for proper p53 activation (Zhang et al., 2009).

In intriguing contrast, growth-promoting conditions, under which p53 is inactivated, are associated with MDM4 phosphorylation at Ser367 by AKT, a key serine/threonine kinase in the PI3K pathway. In this instance, engagement of 14-3-3 increases MDM4–MDM2 multimerization, which in turn stabilizes MDM2. Deregulation of this process poses a risk for pro-survival AKT oncogenic activity (Lopez-Pajares et al., 2008) (refer to Section ‘*Combinatorial MDM4-targeting*’ for a discussion of its therapeutic scope). The *in vivo* significance of MDM4 phosphorylation for its stability was evident in mice and derived MEFs, where substitution of key serines for alanines increased its stability (Wang et al., 2009). Such molecular information offers scope for designing therapeutic regulation of oncogenic MDM4 activities, where particular kinases and the nature of their partners of interaction appear to be critical cell fate determinants.

Additionally, CK1α phosphorylation of MDM4 Ser289 promotes p53 engagement and its inhibition (Wei et al., 2016). Vitally, in a healthy cellular response to DNA stress, interaction between CK1α and MDM4 is disrupted, liberating p53. More precisely, this engagement is interrupted when DNA damage stimulates CHK2 phosphorylation of MDM4 Ser367 (Wu et al., 2012), in a process mediated through ATM (L. Chen et al., 2005; Pereg et al., 2005). Critically, an absence of CK1α and/or MDM4 is p53 activating (Wei et al., 2016) and high CK1α activity would be anticipated to be oncogenic, which is in keeping with recent findings demonstrating CK1α overexpression correlates with poor survival in colorectal cancer (Richter et al., 2018).

p53 activity is also suppressed when MDM4 is phosphorylated at Ser314, leading to stabilization of the MDM2–MDM4 complex and the suppression of p53 activity (as further elaborated in Section ‘*Ubiquitination*.’). MDM4 Ser314 phosphorylation is activated by two tyrosine kinase signaling pathways (Figure 2; as briefly introduced in Section ‘*Wild-type p53*’), which are driven respectively by the human epithelial growth factor 4 HER4 (ErbB4) (Gerarduzzi et al., 2016) and AXL receptor tyrosine kinase (de Polo et al., 2017). Intriguingly, both converge in the activation of CDK4/6 to phosphorylate MDM4 Ser314. In addition, AXL also triggers p38 MAPK that activates MDM4 Ser314 phosphorylation (Figure 2). It will be fascinating to understand how kinase preference is dictated biologically. The oncogenic risk of this process is consistent with elevated AXL levels in numerous tumors, and conversely, that increased chemosensitivity is linked to its suppression, at least in neuroblastoma (de Polo et al., 2017).

#### Ubiquitination

MDM4 levels are regulated in a stress-dependent manner by the E3 ligase activity of MDM2 (Kawai et al., 2003; Pan and Chen, 2003). The RING domain of MDM4 is essential for this regulation during normal growth (Pant et al., 2011). In response to DNA damage (ionizing radiation), the kinase function of ATM directly at MDM4 Ser403 and mediated through CHK2 at MDM4 Ser342, Ser367 dictates MDM4 susceptibility to MDM2-mediated-ubiquitination and degradation (Chen et al., 2005). Further, MDM4 Ser367 phosphorylation, which mediates 14-3-3 binding, enhances its interaction with MDM2 (LeBron et al., 2006) (Section ‘*Phosphorylation/dephosphorylation*.’), which is likely to explain why Ser367 has the most profound influence over MDM4 ubiquitination and stability (Chen et al., 2005). Further, in response to ribosomal stress, MDM4 is degraded in a process involving MDM2 interaction with the ribosomal 60S subunit L11, resulting in p53 activation (Gilkes et al., 2006). In response to oncogenic stress in contrast, ARF binding to MDM2 promotes MDM4 ubiquitination and degradation, independent of MDM4 C-terminal phosphorylation (Li et al., 2012).

In cancer, the normal regulation of MDM4 by MDM2 can be disrupted, leading to accumulation of MDM4 and in turn, the suppression of wt p53 activity. MDM4 ubiquitination can be inhibited by its binding at its RING domain to non-coding 5S rRNA (Li and Gu, 2011). Post-translational modifications of MDM4 may also alter its normal regulation. For example, overexpression or activation of AXL can induce phosphorylation of MDM4 at Ser314. This phosphorylation stabilizes MDM4 against MDM2 degradation and increases the binding avidity between the two proteins driving their nuclear localization (de Polo et al., 2017) (Section ‘*Phosphorylation/dephosphorylation*.’). Pertinently, in tumor cells, nuclear location of MDM4 is reported to predominate, suggesting that its stabilized mislocation is a mode of its deregulation in human cancer (Gembarska et al., 2012; Leventaki et al., 2012). Targeting this protection of MDM4 to direct it for proteasomal degradation has potential for therapeutic application, and a small molecule, camptothecin analog (F118) is demonstrating proof-of-concept. Depending on context, this treatment can induce senescence in the presence of wt p53, or apoptosis in the absence of active p53 (Ling et al., 2014).

In contrast to the potential destructive impact of MDM2-mediated ubiquitination of MDM4, the ubiquitin E3 ligase PELI1 mediates MDM4 ubiquitination, but does not lead to its proteasomal degradation. PELI1-mediated ubiquitination of MDM4 promotes the cytoplasmic localization of MDM4, defining an alternative type of post-translational control that influences cellular outcome. PELI1-mediated MDM4 ubiquitination is linked to the release of wt p53 and its activation. PELI1, through its tight regulation of MDM4 was shown to be critical for proper p53 function. The loss of PELI1 and consequent nuclear MDM4 localization in cutaneous melanoma is attributed to the capacity of these cancers to maintain wt p53, while failing to induce an efficient tumor suppression (Li et al., 2018).

## Oncogenic MDM4 functions

While the earliest oncogenic MDM4 activities identified were demonstrated to be wt p53 dependent, recent findings indicate that its repertoire is far more extensive with ramifications for cancers lacking functional p53 as we discuss in the following section.

### MDM4 inhibits wt p53 transcription in cancers

The capacity of MDM4 to curb wt p53 activity (as discussed in Section ‘*Wild-type p53*’) is exploited in numerous cancers (Karni-Schmidt et al., 2016). Inhibition of p53 transcription and the promotion of its degradation by MDM2 are well-established oncogenic functions of MDM4 in cancers. These oncogenic capacities are frequently achieved through *MDM4* gene amplification (Danovi et al., 2004). Demonstration of increased levels of MDM4 protein, independent of gene amplification, was a groundbreaking discovery that was demonstrated initially in melanoma (Gembarska et al., 2012). The demonstrated efficacy of targeting MDM4 to reactivate wt p53 tumor suppressive activity (Karni-Schmidt et al., 2016) has provided a strong rationale for therapeutic targeting MDM4 in this context, and multiple new approaches are being explored (as discussed in Section ‘**MDM4 therapeutics and as a prognostic biomarker**’).

### MDM4 promotes cancers driven by mutant p53

Importantly, MDM4 has proven to be oncogenic not only in the context of wt p53, but recent work from our group (Miranda et al., 2017) and the Lozano lab (Xiong et al., 2017) also demonstrated that MDM4 promotes cancers in a mutant p53 context, contrary to the prevailing dogma. Specifically, we demonstrated that MDM4 inhibition suppresses the growth of human breast tumor cells with mutant p53 both *in vitro* and *in vivo* (Miranda et al., 2017). In parallel, genetically engineered mice demonstrated the oncogenic power of MDM4 in a mutant p53 knock-in model. MDM4 oncogenic mechanisms that are independent of wt p53 function are necessary to explain these outcomes. At this point, whether MDM4 specifically exacerbates mutant p53, beyond exerting wt p53-independent effects, remains to be demonstrated.

### MDM4 oncogenic functions independent of wt p53

Oncogenic MDM4 functions, independent of wt p53 were convincingly demonstrated in mice models lacking p53. Male mice lacking p53 and overexpressing MDM4 had shorter survival, more tumors and an altered tumor spectrum, compared with their p53 null counterparts (Xiong et al., 2017). Mechanisms responsible for these p53-independent MDM4 activities are not yet fully delineated, but CDKN1B (p27Kip1) (de Lange et al., 2012), RB1 (Hu et al., 2016), and E2F1 (Wunderlich et al., 2004) have been implicated.

Important findings from the Eischen group demonstrate that both MDM4 and MDM2 inhibit DNA break repair. Disruption of the repair response results from MDM4 engagement of Nbs1 of the Mre11–Rad50–Nbs1 (MRN) DNA repair complex in response to DNA damage (Carrillo et al., 2015). While in the context of the additional activities of wt p53, this has important function for controlling the speed of repair to ensure fidelity, in the absence of wt p53, this poses a serious risk for cancer. This offers new direction for therapeutic MDM4 targeting (Eischen, 2017) (see Section ‘*Combinatorial MDM4-targeting*’).

### MDM4 & EMT/migration

MDM4 protein up-regulation was found to correlate with EMT-like transition in both breast and prostate cell lines and clinical samples. This phenotype also correlated with coincident MDM2 down-regulation. Importantly, the molecular signatures of migration, invasion and metastases may be discretely regulated in different cancers and subtypes (Aiello et al., 2018). Of note, however, while correlation between MDM4 with EMT was evident; increased cell migration corresponded only with elevated MDM2 levels, but not with reduced MDM4 levels (Slabakova et al., 2015). Further, in a separate study, when breast cancer samples were stratified for *TP53* mutation, high *MDM4-FL* levels, relative to *MDM4-S* levels, were identified to be coincident with metastatic spread in breast (Grawenda et al., 2015). Together these studies indicate that MDM4 contributes to EMT and metastatic spread, but its contribution to migration remains to be substantiated.

### MDM4 & metabolism

A role for MDM4 in regulating a range of metabolic processes is emerging, and consequently predicting new understanding of how it may be corrupted to become oncogenic, but also in turn be a target for therapy.

#### MDM4 & lipid metabolism

A potential oncogenic role of MDM4 overexpression in lipid metabolism is implied from its influence on the role of p53 in fat accumulation. p53 regulation of lipid metabolism prompted its reputation as the ‘Guardian of Corpulence’ (Bazuine et al., 2009) with refining roles for its regulators MDM4 (Kon et al., 2018) and MDM2 (Liu et al., 2017).

A role for the MDM4–p53 axis in the regulation of lipid metabolism, and thermogenic programs in adipose tissues, was recently exposed through mice models (and to avoid confusion, the human nomenclature for MDM4 will be used, rather than Mdm4 for mice). Specifically, altered lipid metabolism was linked to activated p53 in the absence of MDM4, in response to high calorific intake and reduced temperature. This involved a shift from storage of excess energy in white fat to brown fat in adipose tissue, accompanied by increased lipid oxidation to generate heat in response to cold. The transition was accompanied by an increase in lipid oxidation enzymes (e.g. among others EVOVL3 fatty acid elongase) to support the increased metabolic function (Kon et al., 2018). Importantly, while the effect was p53-dependent, the use of an artificial p53 mutant suggested that full p53 transcriptional competency was not required. The findings were based on an acetylation-deficient p53-3KR (substitution of LYS to ARG: K117R, K161R, K162R) mutant, which retains ferroptosis and DNA-binding capacity, but lacks apoptotic, senescence, and growth arrest capacity. In addition, these mice lacking MDM4 were also protected from insulin resistance (Kon et al., 2018). These studies predict that in the context of elevated MDM4 levels, a consequent inhibition of wt p53 would favor white fat deposition in adipocytes in the context of a high fat diet (prevalent in technologically advanced societies), with diabolical ramifications for the emerging link between obesity and cancer (Deng et al., 2016).

An intriguing parallel study linked reduced p53 activation associated with compromised MDM2 activity to decreased accumulation of lipid and enhanced glucose tolerance in response to a high-fat diet (Liu et al., 2017). This was associated with a loss of RPL11 interaction and consequent failed signaling to p53. It is tempting to speculate that these individual studies glimpse distinct aspects of a larger network of p53 regulation of lipid metabolism. Whether MDM4 and MDM2 can also work in combination, in a p53-dependent manner, to engage these metabolic outcomes will be fascinating to investigate.

The suggestion of targeting these axes to treat diseases associated with obesity is now being considered, i.e. by administering MDM4 inhibitors (Kon et al., 2018) or targeting these MDM2 partnerships (Liu et al., 2017). This work defines MDM4 and MDM2 as regulators of p53-dependent lipid metabolism through their influence on p53 levels and activity. In this context wt p53 is key, where its levels are directly regulated by calorific intake and body temperature.

#### MDM4 & glycolysis

MDM4 evidently regulates glycolysis through negative regulation of p53, involving ubiquitously expressed prefoldin like chaperone (UXT). MDM4 is stabilized through UXT engagement at endogenous protein levels, with consequent reduction in p53 levels and suppression of its transactivation activity. UXT-mediated p53 inhibition results in an activation of NF-κB, leading to induction of glycolysis. Correlation between UXT and cell growth stimulation implicated its oncogenic function, which in turn linked to an increase in the NF-κB signaling pathway (Qi et al., 2015). This defines another potential site for therapeutic intervention.

## MDM4 therapeutics and as a prognostic biomarker

The therapeutic potential of MDM4-based therapies was implied from the proof-of-concept studies in a wt p53 cancer context (See Section ‘*MDM4 inhibits wt p53 transcription in cancers*’). Importantly also, tolerance of the loss of MDM4 in adults (Garcia et al., 2011), contrasts the complications of cytopenias emerging with a first lead MDM2 inhibitor (Nutlin; Burgess et al., 2016). A number of recent reviews are dedicated to emerging MDM4-based treatments (Marine and Jochemsen, 2016; Bardot and Toledo, 2017; Tisato et al., 2017), and thus our focus will be on recent advances.

**Figure 3:**
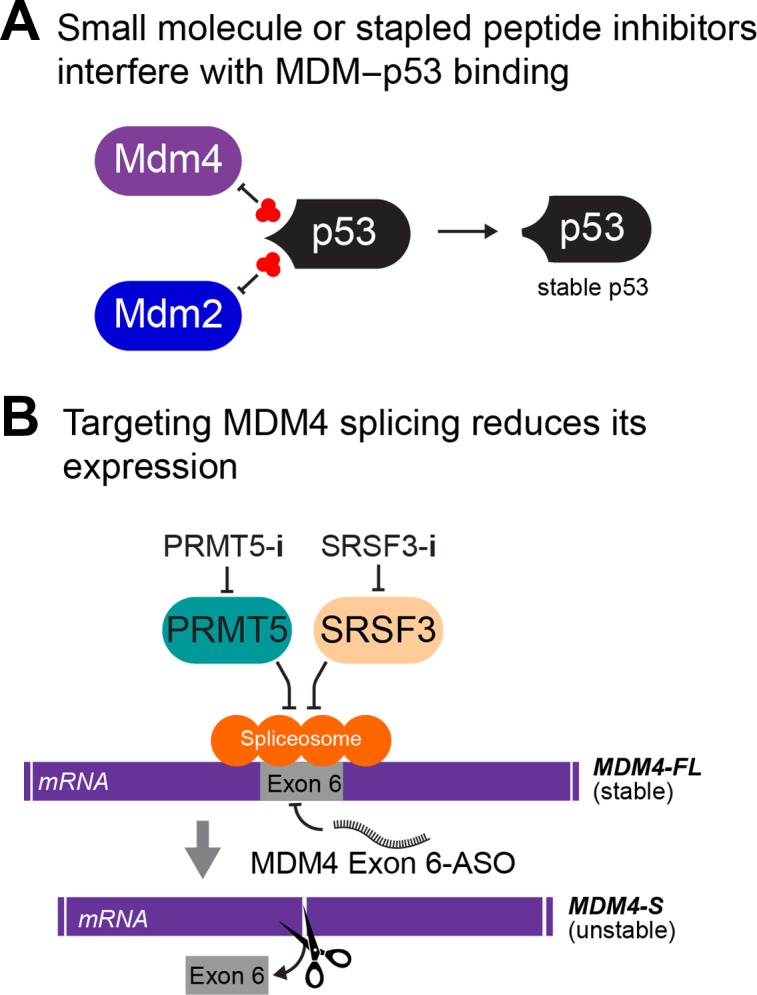
MDM4 is an emerging therapeutic target in cancer. Two primary approaches are to (**A**) interrupt its engagement of p53 using small molecules and stapled peptides and (**B**) reduce MDM4 expression levels by interfering with its splicing to force the expression of its unstable isoform MDM-S.

Structural elucidation of the interface between wt p53 transactivation domain and MDM2 N-terminus (Kussie et al., 1996) fostered rational approaches to interrupt p53 engagement of MDM2, and also its structural homolog MDM4. There are two main types of MDM4-targeting therapies in current development: either to inhibit MDM4–p53 protein using small molecules and stapled peptides or to interfere with the MDM4 expression, largely by targeting RNA splicing factors (Figure 3).

### MDM4 inhibition

Potent MDM4 targeting using dual MDM2 and MDM4 inhibitors was demonstrated with the first-generation MDM2 prototype inhibitor Nutlin-3a, a cis-imadazoline analog identified and developed by Vassilev et al. (2004). In spite of its significantly lower affinity toward MDM4 than toward MDM2, the near absence of MDM2 in a retinoblastoma model with elevated MDM4, resulted in remarkable response when the drug was administered directly to the tumor site (Laurie et al., 2006). Although the unfortunate side effects of Nutlin-3a halted its clinical development as a systemic drug, a new generation of dual inhibitors are in the pipeline (Burgess et al., 2016) with modifications to enable coupling to bioactive carriers to direct delivery (Twarda-Clapa et al., 2017) or reduce side effects.

Dual MDM2–MDM4 inhibitors from the class of stapled-peptides are being avidly developed. Stapled-peptides are based on chemical ‘stapling technology’ that introduces a hydrocarbon linker between two non-adjacent amino acids in a peptide (Bernal et al., 2010) and consequently increases robustness in biological systems. The stapled α-helical peptide ALRN-6942 from Aileron Therapeutics is a dual inhibitor that has equal binding potency for MDM2 and MDM4 and is currently in clinical trials in a wt p53 setting (Carvajal et al., 2018), where it is well tolerated and showing early indications of antitumor efficacy in hematological and solid malignancies (NCT02264613, NCT02909972; Tisato et al., 2017). Clinical trials were established on its success in preclinical models. An example of its pre-clinical success is in acute myeloid leukemia models (AML), where MDM4-FL is overexpressed. ALRN-6942 efficacy was attributed to its cell membrane penetrance capability, together with its high affinity for both MDM2 and MDM4, which frees p53 to selectively inhibit cancer cell growth inhibition, while sparing from general toxicity (Carvajal et al., 2018).

Other groups are also developing dual MDM2 and MDM4 inhibitor prototype, stapled peptides, but these are yet to enter clinical trials. While these compounds are based around the sites of p53–MDM2 engagement, progressive refinements from the early compound SAH-p53-8 (Bernal et al., 2010) have improved features of increased target affinity (STAPLINs; Tan et al., 2015); cellular uptake (ATSP-7041; Uldrijan et al., 2007; Chang et al., 2013) and improved activity and specificity (sMTide02 and sMTide2A; Brown et al., 2013).

Structural modeling of MDM2 and MDM4 is fundamental to the improved design of staple peptide dual inhibitors. Despite ~60% N-terminal structural identity between MDM2 and MDM4, there is significant distinction between their binding pocket conformation and dual inhibitors are proving challenging to design. To overcome the pocket distinctions and improve dual inhibitor efficiency, two new strategies are proposed: to impose upon MDM4 Tyr100/99 (where Tyr99 is the key residue for p53 engagement described in Section ‘*Phosphorylation/dephosphorylation*.’) to stabilize MDM4 into an open state or to develop designs that are not affected by the conformation of these critical residues (Lee et al., 2017). In another study, adjacent MDM4 residue Leu98 was also identified for its determining role in staple-peptide engagement (Chee et al., 2017). Together, these modeling studies highlight the importance of this region for p53 engagement and emphasize the benefit of structural elucidation for optimizing drug design. Future improvements to extend the feasibility of these therapies are expected to focus on administration, through a route other than intravenous, on improved cell penetration, and on the spectrum of molecular targets engaged. Interactors beyond wt p53 are likely to be relevant in a mutant p53 context (relevant to Section ‘*Mutant p53*’).

Specific, small molecule inhibitors of the MDM4–p53 interaction remain an aspiration that has not advanced to the clinic. The developed prototype, SJ-172550, was identified in a small molecule screen by the Dyer group (Reed et al., 2010), no doubt inspired by their promising findings in retinoblastoma with Nutlin-3a. The compound binds to MDM4 and induces a conformation incapable of sequestering p53. Issues with stability and mechanistic complexity however have halted further development of this compound (Bista et al., 2012). Similarly, other small molecule inhibitors of MDM4 are inadequate for translation to patients and remain as laboratory tools only, including XI-011 (Roh et al., 2014) and NSC207895 (Wang et al., 2011).

### Targeting MDM4 mRNA generation

Targeting MDM4 alternative splicing has arisen as a topical approach for cancer therapies in a wt p53 context. The underlying concept is to inhibit the inclusion of MDM4 exon 6, to prevent formation of stable *MDM4-FL*, which inhibits wt p53 (Section ‘*Wild-type p53*’). Inhibition of the protein arginine methyltransferase, PRMT5, which targets the spliceosome (Bezzi et al., 2013; as introduced in Section ‘*MDM4 isoform splicing*’), is being actively developed as a means to activate wt p53 function (Gerhart et al., 2018). The capacity of PRMT5 inhibitors (such as GSK3326595) to alter MDM4 splicing is a major therapeutic focus. Although p53 mutation proved the most frequent indicator of resistance to PRMT5, this was by no means a strict determinant. As PRMT5 has multiple additional targets, some of these are also likely to contribute to its effects and treatment success. Future studies will be required to properly tease apart the relevant targets pertinent to the cancer inhibitory function of current PRMT5 inhibitors (Gerhart et al., 2018).

Direct targeting of the splicing factor SRSF3, which is instrumental in exon 6 inclusion (described in Section ‘*MDM4 isoform splicing*’), is a mode of inhibition being explored, using small molecule (TG003) targeting of its activating kinase (CLK). Another key approach under development is anti-sense oligonucleotide (ASO) therapy, that causes ‘skipping’ of MDM4 exon 6 with a consequent generation of unstable MDM4-S. This proved to be efficacious in wt p53 melanoma mouse models (Dewaele et al., 2016). The recent flurry of interest in ASO-based therapies augurs well for the clinical application of MDM4 ASO-therapies (Marine and Jochemsen, 2016).

### Combinatorial MDM4-targeting

Potential for MDM4 combinatorial targeting is attractive although largely remaining at the pre-clinical level. Specific examples include the combined depletion of MDM4 and protein kinase K to treat drug recalcitrant forms of metastatic uveal melanoma (Heijkants et al., 2018), in a wt p53 context. Also in a wt p53 context, treatment of castrate resistant prostate cancer has been proposed using the combination of a small molecule inhibitor of MDM4 expression (NSC207895) and a MDM2 inhibitor (Nutlin-3). The rationale for this approach is that co-expression of MDM2 and MDM4 leads to stabilization of androgen receptor (AR) and further, that MDM4 modulates MDM2-mediated AR ubiquitination. At least *in vitro*, combined treatment activates p53 and destabilizes AR in prostate cancer cells (Chopra et al., 2018). Another compound with demonstrated potential in prostate cells to target the MDM2–MDM4 axis is the natural MDM2 inhibitor Inulanolide A (InuA). InuA binds to the ring domains of these proteins, disrupting their engagement and promoting MDM2 degradation. Further, it also binds to the DNA binding domain of NFTA and causes the inhibition of MDM2 transcription. Pertinently, this molecule demonstrated activity against both wt p53 and mutant p53 prostate cancer cells, regardless of their AR status (Qin et al., 2017).

Building on from the identification of possible oncogenic AKT growth involving MDM4 Ser367 phosphorylation driving p53 depletion (Section ‘*Phosphorylation/dephosphorylation*.’), PI3K inhibitors are being explored. Consistently, PI3K inhibitor LY294002 reduces MDM4 levels in a range of cancer cells with wt p53 (U2OS, LNCap, and OVCA420), associated with an increase in p21 levels (Lopez-Pajares et al., 2008).

Correlation between cisplatin resistance and elevated MDM4 expression levels, in non-small cell lung cancer, also predicts the scope for combined MDM4 inhibition in this disease (Zhao et al., 2017). Estrogen receptor (ER)–MDM4–MDM2 combination targeting luminal A/B breast cancer subtypes is also offering possibilities due to the association between these targets, independent of p53 status. Specifically, ERα can up-regulate the expression of MDM4 and MDM2 in some breast cancers and these effects can be blocked by endocrine therapy fulvestrant and tamoxifen (Swetzig et al., 2016.)

The original finding that MDM4 and MDM2 retard the DNA repair process (Section ‘*MDM4 oncogenic functions independent of wt p53*’) further opens new avenues for cancer therapy. Promoting DNA repair while inhibiting MDM4 and MDM2 is therefore rational (Eischen, 2017). Additional stimulation of wt p53 function in the context of this combination is also sound.

## Conclusions

The growing interest in the biochemical and biological functions of MDM4, both p53 dependent and independent, is revealing it as a key regulator of vital cellular processes. Importantly, the dysregulation of MDM4 has significant impacts on multiple hallmarks of cancers, often resulting in compromised tumor suppression and cancer development. Most keenly evident from this review is the vital role of MDM4 C-terminal RING domain for engaging p53 RNA (Malbert-Colas et al., 2014) and also MDM2, while its N-terminus is instrumental in the primary engagement of the p53 protein (Popowicz et al., 2008). The generation of multiple isoforms of MDM4 to some extent also provides regulation at the level of expression. The understanding of the function and expression of MDM4 isoforms is only partial, and potentially results in confusion. There is a clear need for molecular tools to facilitate the study these isoforms, and their identification in the clinic. The poor tolerance of MDM2 therapeutics, together with the emerging relevance of MDM4 as an attractive therapeutic target, renders the development of MDM4 specific inhibitors ever more important and timely.
