# Corrigendum to ‘The long and the short of it: the MDM4 tail so far’

**DOI:** 10.1093/jmcb/mjz109

**Published:** 2019-12-26

**Authors:** Sue Haupt, Javier Octavio Mejía-Hernández, Reshma Vijayakumaran, Simon P Keam, Ygal Haupt

**Affiliations:** 1 Tumor Suppression Laboratory, Peter MacCallum Cancer Centre, 305 Grattan Street, Melbourne, Victoria 3000, Australia; 2 Department of Clinical Pathology, The University of Melbourne, Parkville, Victoria 3010, Australia; 3 Department of Biochemistry and Molecular Biology, Monash University, Clayton Campus, Victoria 3800, Australia; 4 Sir Peter MacCallum Department of Oncology, The University of Melbourne, Parkville, Victoria 3010, Australia

In this article (p.240, *MDM4 Inhibition* section, second paragraph), the drug name of Aileron stapled peptide ‘ALN-6924’ was incorrectly cited (as ‘ALN-6942’). This mistake was due to human error. The conclusions of the review are not affected and the authors apologize for this error.

The corrected text is shown below:

Dual MDM2–MDM4 inhibitors from the class of stapled-peptides are being avidly developed. Stapled-peptides are based on chemical ‘stapling technology’ that introduces a hydrocarbon linker between two non-adjacent amino acids in a peptide (Bernal et al., 2010) and consequently increases robustness in biological systems. The stapled α-helical peptide ALRN-6924 from Aileron Therapeutics is a dual inhibitor that has equal binding potency for MDM2 and MDM4 and is currently in clinical trials in a wt p53 setting (Carvajal et al., 2018), where it is well tolerated and showing early indications of antitumor efficacy in hematological and solid malignancies (NCT02264613, NCT02909972; Tisato et al., 2017). Clinical trials were established on its success in preclinical models. An example of its pre-clinical success is in acute myeloid leukemia models (AML), where MDM4-FL is overexpressed. ALRN-6924 efficacy was attributed to its cell membrane penetrance capability, together with its high affinity for both MDM2 and MDM4, which frees p53 to selectively inhibit cancer cell growth inhibition, while sparing from general toxicity (Carvajal et al., 2018).

